# Quality of Life and Chemosensory Function in Post-COVID Patients: Adherence to Olfactory Training

**DOI:** 10.1177/00034894251396189

**Published:** 2025-12-16

**Authors:** Stephanie Wiederstein, Stefan Grasl, Verena Rappold, Johanna Stueckler, Roswitha Schneeberger, Christian A. Mueller, Bertold Renner

**Affiliations:** 1 Department of Otorhinolaryngology – Head and Neck Surgery, Medical University of Vienna, Austria; 2 Rehabilitation Clinic Tobelbad, Allgemeine Unfallversicherungsanstalt (AUVA), Graz, Austria; 3 Institute of Experimental and Clinical Pharmacology and Toxicology, Friedrich-Alexander-Universität Erlangen-Nürnberg, Germany; 4 Institute of Clinical Pharmacology, Medical Faculty Carl Gustav Carus, Technische Universität Dresden, Germany

**Keywords:** coronavirus, olfactory dysfunction, quality of life, olfaction, olfactory training, adherence

## Abstract

**Objective:**

To evaluate adherence to and effects of standardized olfactory training (OT) after COVID-19 infection.

**Methods:**

In this prospective study, 475 patients (mean age 47.4/SD 9.5 years) with olfactory dysfunction (OD) after COVID-19 infection participated in OT on average 13 months (range 0-31 months) post-infection. Patients were assessed 3 times with an average interval of 3 months using questionnaires on quality of life (QoL), chemosensory functions, and OT adherence.

**Results:**

Overall, 363 patients (76.4%) took part in the first 2 and 49.1% in all 3 testing sessions. Before the initial assessment, 32.8% had performed OT in the past without any instructions, so that merely 9.1% adhered to recommended standards. By the second evaluation, after standardized OT was introduced to the participants, 31.1% (n = 106) reported following the training as recommended, decreasing to 18.5% (n = 43) by the third assessment, indicating that most participants did not complete the recommended duration of at least 12, preferably 24 to 36 weeks of olfactory training. QoL and subjective olfactory acuity significantly improved between the first and following testing sessions (*P* < .001), but these improvements were not associated with adherence to OT. However, male sex (*P* = .035), non-medical profession (*P* = .025), increased age (*P* = .009), and reduced QoL (*P* = .027) were observed factors for advanced training adherence to recommended standards.

**Conclusion:**

Overall, only a small proportion of participants consistently followed OT. Higher adherence, however, was associated with male sex, non-medical profession, older age, and greater impairment in QoL. Nevertheless, on average there was a clear improvement in QoL and subjective sense of smell over the testing period.

## Introduction

Since the outbreak of the severe acute respiratory syndrome coronavirus 2 (SARS-CoV-2) disease in 2019, over 775 million cases have been reported to the World Health Organization (WHO).^1^ Clinical presentations range from asymptomatic cases to severe respiratory complications.^2,3^ Olfactory dysfunction (OD), encompassing anosmia, hyposmia, parosmia, and phantosmia, is an early and distinctive indicator of infection. This is noteworthy because OD can manifest itself even in otherwise asymptomatic individuals, making it valuable for screening and diagnosis purposes in COVID-19 cases.^4-6^ Approximately 50% to 75% of COVID-19 patients develop OD,^5,7-9^ with a higher prevalence among younger individuals, females, and those with milder cases of the disease.^5,10-12^ With more recent virus variants like omicron or delta, olfactory disturbances occur less frequently, with omicron averaging around 13%.^13,14^ The cause of OD in COVID-19 remains unclear, but may involve olfactory cleft edema, injury to the olfactory epithelium, and damage to the olfactory bulb.^4^ Edema in the olfactory cleft might hinder odorant delivery, despite nasal congestion being less common in COVID-19 cases.^15,16^ Injury to olfactory epithelial cells and ACE2 receptors on supporting cells may contribute to decreased sensitivity and odorant receptor loss.^4,17^ Retrograde axonal transport of SARS-CoV-2 to the olfactory bulb and central nervous system has been suggested, supported by animal studies and neuroimaging findings.^4,18,19^ Further research is essential to better comprehend and address OD as a symptom of COVID-19.^4^

Multiple studies have examined recovery rates and risk factors for persistent OD post-COVID-19, using surveys or objective tests. Initially, rapid recovery with complete resolution within about 10 days was reported.^4,20,21^ However, subsequent research suggests that self-reported recovery may be exaggerated. Discrepancies between self-reports and objective evaluations have been noted.^22^ Boscolo-Rizzo et al conducted a case-control study with a 401-day follow-up, finding OD in 46% of cases and 10% of controls, with 7% of COVID-19 cases being anosmic.^23^ Considering COVID-19’s global impact, many patients will still endure severe OD despite reported high recovery rates.^4^

Previous studies have established that OD significantly diminishes individuals’ quality of life (QoL) and increases the risk of depression and anxiety.^5,24-26^ OD also disrupts social interactions and dietary habits, as affected individuals often lose enjoyment in eating and may consume less. The inability to detect hazardous odors like smoke or gases can pose life-threatening risks, making therapies that enhance smell crucial.^24,27,28^ Olfactory training (OT) involves daily exposure to 4 odors for 20 to 30 seconds each, twice a day for at least 12 weeks.^29,30^ The objective is to stimulate the regeneration of olfactory neurons and subsequent neuronal plasticity by increasing neurotrophic factors through continuous stimulation of the olfactory receptors.^5,31-33^ Numerous studies have demonstrated that OT is the most effective treatment for post-viral olfactory dysfunction (PVOD), with minimal risks. Additionally, it is recognized that the sooner OT is initiated following infection or onset of OD, the more favorable the outcomes, with current evidence recommending initiation within the first 12 months.^34-36^ Nevertheless, treatment adherence poses a challenge in certain studies.^37,38^ Consequently, our investigation aimed to study the adherence and compliance in patients with possible treatment-related restrictions according to insurance settings, along with potential influencing effects.

## Materials and Methods

### Study Cohort

**Figure 1.**
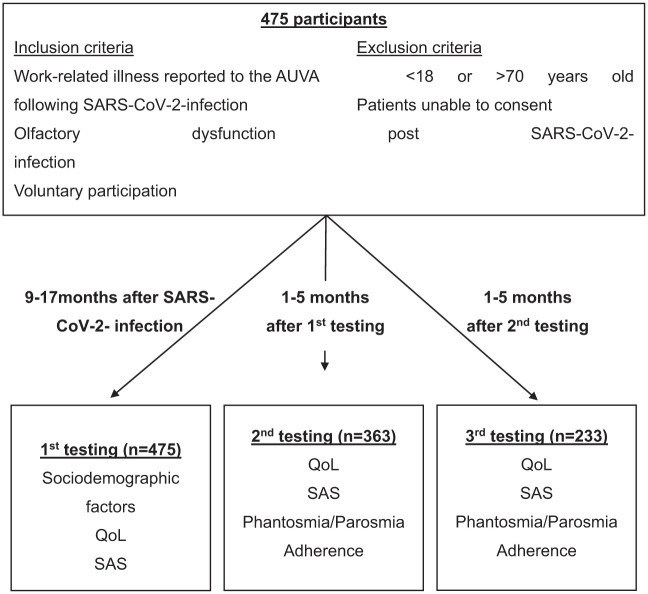
Flowchart of the testing procedure (AUVA, SARS-CoV-2, QoL, SAS). AUVA, Allgemeine Unfallversicherungsanstalt; SARS-CoV-2, severe acute respiratory syndrome coronavirus type 2; SAS, self-assessment of smell; QoL, quality of life.

This prospective study, conducted at the “Allgemeine Unfallversicherungsanstalt” (AUVA, an insurance company for occupational disease), at the Department of Otorhinolaryngology at the Medical University of Vienna, and at the Department of Pharmacology of the Technical University Dresden from January 2021 to January 2023, focused on 475 patients experiencing olfactory disorders following COVID-19 infection confirmed by PCR testing (Figure 1). Patients were recruited for by AUVA and were employed or insured in healthcare facilities. Inclusion criteria were written informed consent, ages 18 to 70, work-related illness recognized by AUVA following SARS-CoV-2 infection, and OD post-infection. Participants reported via questionnaire no smell impairment before COVID-19 infection. OT began on average 12.8 months post-infection (range 0-31 months), with some participants receiving training before their initial assessment.

### Olfactory Training (OT)

OT devices, resembling lipstick containers, contained essential oils (rose geranium, lemon, cajeput, frankincense; Farfalla Essentials AG, Uster, Switzerland), which are absorbed through the air. Approximately 10 to 13 drops of essential oil were dripped onto the inner cotton swab. According to German guidelines and previous studies, patients should smell 4 odors twice daily for about 30 seconds each over at least 12 weeks, preferably 24 to 36 weeks, with devices replaced every 3 to 6 months.^39-41^ The study aimed to examine olfactory training rather than a specific product. The odors were selected by our study partners at the AUVA based on their aromatherapy expertise and established components.

### Data Assessment

Data was gathered at 3 intervals ~3 months apart using questionnaires. Sociodemographic factors such as age, sex, occupation, comorbidities, smoking status, and medication, alongside current taste and smell symptoms, were recorded. Patients assessed the impact of smell and taste disorders on their QoL using a Likert scale ranging from 1 (not at all) to 10 (severe). Self-assessment of odor, taste, and flavor perception used a Likert scale from 1 (no perception) to 10 (excellent perception), including occurrences of parosmia or phantosmia. Training adherence (duration, frequency, and consistency) was evaluated. Compliance was defined as OT engagement at least twice a day for 2 minutes each session.

### Statistics

Statistical analysis was conducted using SPSS Version 23.0 software (IBM Corp, Armonk, NY, USA). Results are presented as mean ± SD. Analysis of variance (ANOVA) was employed to compare QoL and self-assessed chemosensory functions, including parosmia and phantosmia, across different testing periods. Cross-tabulation and chi-square tests analyzed training adherence and its association with potential influencing factors like sex, profession, age, parosmia, and phantosmia. A *T*-test for independent samples demonstrated the association between QoL and OT adherence. All tests were 2-sided, with *P* values below .05 considered statistically significant.

### Ethics

The study was approved by the Ethics Committee of the Medical University of Vienna under EC No 1884/2021 with annual extension. All participating patients received comprehensive information about the study and provided written informed consent. Patients could withdraw at any time without disadvantage.

## Results

This cohort included 475 patients, comprising 360 (75.8%) females and 106 (22.3%) males, with 9 missing sex data points. The mean age was 47.4 ± 9.5 years. The average time from COVID-19 infection to symptom onset was 7.3 ± 33.7 days, with 361 (82.4%) patients experiencing sudden onset and 77 (17.6%) gradual onset of symptoms, with 37 missing data points. The interval between infection and the initial examination was 12.8 ± 4.3 months (range 0-31). Among the patients, 363 (76.4%) completed both the first and second session, and 233 (49.1%) completed all 3 study visits, with an average of 2.98 ± 1.78 months between the first and second testing and 3.16 ± 1.89 months between the second and third testing. In the first testing, 173 (43.5%) participants reported seeking medical help for chemosensory symptoms after COVID-19 infection, while 255 (56.5%) did not seek medical help, and 77 did not respond.

During the study, 226 testing subjects were employed in medical professions, whereas 232 worked in non-medical fields. Among those in medical professions, 70.4% were registered nurses, nursing assistants, or physicians, while the remaining 29.6% included radiology technicians, paramedics, cleaning staff, occupational therapists, speech therapists, and physiotherapists. Seventeen participants did not provide professional information. In the initial testing, 68 (14.6%) individuals were current smokers, 163 (35%) were former smokers, and 235 (50.4%) had never smoked, with 9 missing smoking status data points.

### Subjective Assessment of Quality of Life and Self-Reported Chemosensory Function

**Figure 2.**
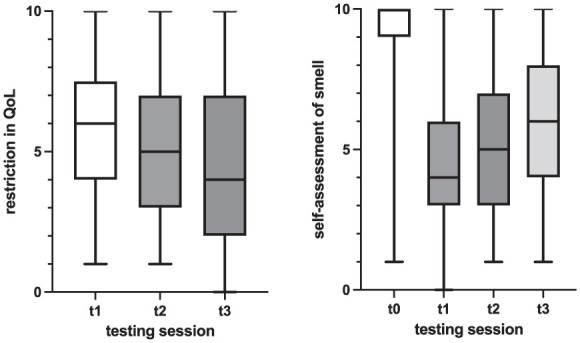
Subjective assessment of smell and restriction in QoL over time (boxes [1, quartile-3, quartile]) represent the middle 50% of data and the inside horizontal lines mark the median value, the 2 horizontal lines mark the whiskers (lower horizontal line = 1, quartile −1.5 interquartile range; upper horizontal line = 3, quartile +1.5 interquartile range).

The mean subjective perception of the decline in QoL due to impaired chemosensory functions was 5.63 ± 2.37 initially, 4.85 ± 2.55 in the second testing, and 4.51 ± 2.66 in the third testing (Figure 2). This indicates progressive reduction in restriction, signifying an enhancement in patients’ QoL over time. Statistical analysis revealed significant differences in the mean QoL scores between the first and second testing, and the first and third testing (*P* < .001). Between the first and second testing, 62 participants (18.4%) reported improved QoL, while 262 (78%) reported no improvement, and 12 (3.6%) reported no change, having rated their QoL as the highest possible score initially. Similarly, between the second and third testing, 40 participants (17.9%) reported improvement, 180 (80.3%) reported no improvement, and 4 (1.8%) had previously given the highest possible score.

In the initial testing, participants rated their sense of smell before contracting COVID-19 at an average of 9.38 ± 1 out of 10. However, the current odor was rated lower, at 4.47 ± 2.16 in the first, 5.46 ± 2.42 in the second, and 5.92 ± 2.47 in the third testing. Significant differences in current odor were observed between the first and second testing, and the first and third testing (*P* < .001). Between the first and second testing, 212 participants (60.4%) reported improvement in subjective olfactory function, while 139 (39.6%) reported no change. Similarly, between the second and third testing, 112 participants (48.7%) reported improvement, 116 (50.4%) reported no change, and 2 (0.9%) had previously given the highest possible score. Regarding olfactory disorder type, 65% (n = 306) reported parosmia and 39.4% (n = 179) reported phantosmia in the first testing. In the second testing, these percentages were 62.4% (n = 224) for parosmia and 38.8% (n = 138) for phantosmia, and in the third testing, 57.9% (n = 135) reported parosmia and 30% (n = 69) reported phantosmia. However, no statistically significant differences were found between the individual testing sessions.

### Olfactory Training Adherence

**Figure 3.**
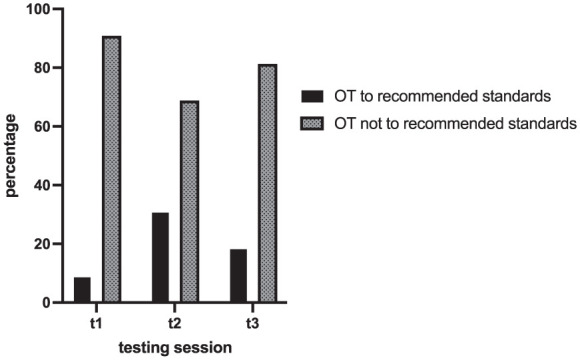
Adherence to OT according to recommended standards over time. OT, olfactory training.

Before the initial testing, 32.8% (n = 156) of participants had engaged in OT, but only 9.1% (n = 43) followed the recommended standards, performing it twice daily for at least 2 minutes. In the second testing, when participants were already enrolled in the study’s OT program, 31.1% (n = 106) adhered to the recommended standards, decreasing to 18.6% (n = 43) in the final testing (Figure 3).

### Factors Influencing Olfactory Training

In the third testing, a significant positive association between being male and following the recommended OT was found (*P* = .035). Twenty-eight point six percent of men followed the OT as recommended, compared to 15.5% of women. Additionally, 26 participants (23.6%) employed in non-medical professions adhered to the OT guidelines, whereas only 14 (12.2%) of those in medical professions followed the standard protocol. Among the 40 participants adhering to the OT guidelines in the third testing, 26 (65%) were in non-medical professions and 14 (35%) were in medical professions. The chi-square test indicated a significant association between non-medical occupation and higher OT adherence (*P* = .025).

A statistically significant association was identified between age and adherence to the recommended OT protocol in both the second and third tests. During the second testing, 48 individuals (14.6%) were aged 35 or younger, 217 (66%) were between 36 and 55 years old, and 64 (19.5%) were older than 55 years. Among those aged 35 or younger, 16 subjects (33.3%) followed the OT guidelines. In the 36 to 55 age group, 58 (26.7%) adhered to the standard, and among those over 55 years old, 30 (46.9%) adhered to OT. In the third testing, 28 individuals (12.3%) were 35 years or younger, 156 (68.7%) were between 36 and 55 years old, and 43 (18.9%) were older than 55 years. Among the youngest group, 4 subjects (14.3%) adhered to the OT guidelines, while 23 (14.7%) from the middle age group and 15 subjects (34.9%) from the oldest group followed the recommended training. This indicates a positive association between older age and higher adherence to training (*P* = .009).

### The Influence of Quality of Life and Subjective Olfactory Function on Olfactory Training

In the first and third testing, there was no significant difference in QoL between subjects who adhered to the recommended OT and those who did not (*t*[449] = −0.777, *P* = .437; *t*[226] = −1.146, *P* = .253). However, during the second testing, a significant difference in QoL was evident between subjects who followed the standard OT regimen and those who did not. On average, there was a 0.66-point difference in QoL, with participants who adhered to the recommended training reporting a greater restriction in QoL (95% CI [−1.253 to −0.074], *t*[331] = −2.215, *P* = .027).

There was no significant difference in subjective olfactory ability between subjects who adhered to the recommended OT and those who did not in the first (*t*[461] = 0.391, *P* = .696), second (*t*[336] = −1.305, *P* = .193), nor third testing (*t*[231] = 1.598, *P* = .111).

## Discussion

This study aimed to assess adherence to and effects of standardized OT following COVID-19 infection. The primary outcome revealed a notable lack of adherence to OT. Among 475 participants who commenced OT, just over three-quarters attended the second testing, and fewer than half completed the third. At the second assessment – 12 weeks after study initiation, corresponding to the recommended minimum training duration – 31.1% of participants adhered to the prescribed regimen. By the third assessment, 6 months after the start of training, adherence had decreased to 18.6%. This aligns with previous research, indicating a marked decline in adherence to OT over time.^37,38,40^ Non-adherence to prescribed treatments is widely recognized in the medical field. Numerous studies highlight that patients neglect to adhere to their prescribed medications in 50% of instances, with rates soaring to 80% for asymptomatic conditions like arterial hypertension.^42-44^

Our study identified a statistically significant link between OT adherence and sex, with men adhering more than women, consistent with previous research suggesting that women’s caregiving roles may limit time for self-care.^37,45,46^

We also observed a statistically significant association between OT adherence and occupation in the medical field. In the final assessment, 23.6% of individuals in non-medical professions adhered to OT standards, compared to 12.2% in medical professions. To our knowledge, no previous studies have explored the impact of occupation on adherence to OT protocols. Irregular schedules, night shifts, and caregiving demands in medical professions may hinder establishing a consistent OT routine.

Unlike Fornazieri et al, we observed a notable association between OT adherence and participant age.^37^ Participants were categorized into 3 age groups. In the second assessment, approximately one-third of the youngest group (35 years and younger) and just under one-quarter of the middle-aged group (between 36 and 55 years) adhered to recommended OT, while nearly half of the oldest group (over 55 years) exhibited adequate adherence to the training. In the third assessment, adherence was just under 15% for the 2 younger groups but slightly over one-third for the oldest group. Literature suggests that medication adherence tends to increase with age, possibly because older patients are more conscious of non-adherence consequences.^45-47^ However, in our context, focusing on daily OT rather than medication intake, which requires consistent time and routine, lower adherence among younger individuals (<56) could be attributed to time constraints due to work or family commitments.

We also explored participants’ subjective QoL as a potential factor influencing training adherence. In the second testing, we observed a significant association between reduced QoL and adherence to OT. Interestingly, individuals adhering to the recommended OT reported a greater decline in QoL, likely because those perceiving a higher impact on their QoL due to OD were more motivated to engage in OT to alleviate their symptoms. However, this association was only observed in the second testing, with a minor QoL difference of 0.66 points on a 10-point scale, indicating limited clinical significance.

Consistent with Haas et al and Schepens et al, we found no statistically significant difference in subjective olfactory function between individuals adhering to the recommended OT and those who did not.^38,48^ Participants who did not follow the recommended training regimen still engaged in some form of OT, albeit less consistently, possibly benefiting from it. Haas et al proposed that the similar lack of adherence in both groups could be attributed to differing patient perceptions: those who perceive improvement in olfactory function may become satisfied and cease training, while those not experiencing improvement may become dissatisfied and skeptical of the efficacy of OT and discontinue training.^38^

Another significant finding of our study is the statistically significant improvement in QoL and subjective olfactory function among participants, particularly between the first and second testing. Improvements between the second and third testing were not significant. However, these results indicate a positive trend in QoL and olfactory function over time. It is important to note that, since our study lacked a control group that did not undergo OT, we cannot establish a causal relationship between OT and the observed improvements. Nonetheless, consistent OT has been shown to have a positive effect on olfactory function in existing literature, thus it is strongly recommended for patients experiencing OD following SARS-CoV-2 infection.^12,49,50^

At the initial assessment, 65% of participants reported parosmia and 40% phantosmia, consistent with previous findings that parosmia is more common post-COVID-19.^2,51^

Our research underscores OT’s efficacy for post-SARS-CoV-2 OD, consistent with previous studies. However, it also reveals significant non-adherence to therapy. Enhancing patient awareness about the importance of OT is crucial This can be accomplished through regular phone consultations, frequent in-person meetings to support and supervise OT progress, and diversifying scent options over time.^37^

Our study has certain limitations. Without a control group, interpreting the causality between OT and improvements in QoL and subjective olfactory function requires caution. Since OT was already an additional therapeutic offering provided by the AUVA, and additional objective chemosensory tests were not feasible due to professional regulations, only subjective olfactory function was assessed through questionnaires. Only a few participants used the offered objective olfactory assessment at the Department of Otorhinolaryngology, Medical University of Vienna. However, previous studies highlight a notable divergence between subjective and objective olfactory function, questioning conclusions based solely on subjective reports.^22^ Additionally, only about half of the 475 initial participants attended all 3 testing sessions, with unknown dropout reasons, introducing potential selection bias.

## Conclusion

This study is the first to evaluate adherence to standardized OT post-COVID-19 infection and its influencing factors within a large patient cohort. Despite OT’s proven efficacy, adherence to recommended OT was low. We observed better adherence associated with male sex, non-medical professions, older age, and greater impairment in QoL. Despite low training adherence, significant improvements in QoL and olfactory function were observed over the study period. Therefore, enhancing patient adherence to OT through awareness is crucial for better olfactory rehabilitation outcomes.
